# Expert Opinion

**Published:** 1920-03-27

**Authors:** 


### SCRAPPING THE TRAINED NURSE.—III.

### Expert Opinion.

In order to ascertain the attitude of the medical profession toward the Regulations of the Ministry of Health, which prevent Trained Nurses from becoming Health Visitors unless they abandon their employment and start out anew as students, I sought an interview with Dr. George Peacocke, one of Dublin's prominent physicians, a gentleman who all his life has been intimately associated with hospital practice and procedure.

Doctor Peacocke, a son of the late Archbishop of Dublin, Chairman of the Irish Branch of the College of Nursing, and a member of the College Executive at headquarters, was at once deeply interested in the question, and expressed amazement at the conclusions arrived at by Dr. Addison and the Board of Education.

It happens that at present there is no Health Ministry in Ireland. Normally, the Chief Secretary for Ireland is the Irish Minister of Health, and the Irish Health Council is at present engaged in pioneering the way to legislation. An Act of Parliament is a necessary preliminary to the setting up of working machinery and the obtaining of funds to carry on the work, and, naturally, the leading men and women in the medical and nursing world are on the *qui vive*.

It is in view of probable forthcoming developments in Ireland that Dr. Peacocke regards the action of the Minister of Health in Great Britain with profound interest.

### The Nurse and the Schoolwoman.

Concerning the qualifications of the Nurse for the post of Health Visitor, Doctor Peacocke's opinion is uncompromising. "There is no comparison," he exclaimed, "between the value of a Trained Nurse for such work and that of a woman who has received training only in a Polytechnic Institute. The nurse has spent three years in the study of disease in all its aspects and the practical nursing of men, women, and children. She has learnt to be kind and sympathetic, and has obtained an insight into the social conditions and surroundings of the homes of her patients."

"In all nurse training schools," he continued, "she receives lectures on Nursing, Medicine, and Surgery, as well as on most of the subjects included in the curriculum required for Health Visitors."

I inquired as to the additional training.

Doctor Peacocke affirms that this could easily be provided in a Hospital. A course of three, or at most six, months' training, he says, in Maternity, Infant, and Child Welfare, and Social problems would be ample additional training in his opinion for any trained nurse.

"Let me put it in another way," said Doctor Peacocke. "To qualify for the post of Medical Officer of Health it is necessary in the first instance to be a registered medical practitioner. Why should there be any difference in the case of a Health Visitor? The Nurses' Registration Act will soon be in operation, and it seems to me that none should be eligible for the post of Health Visitor whose name is not on the Nurses' Register."

"Nurse training schools," Dr. Peacocke continued, "are now making arrangements to appoint Sister-Tutors or Teaching Sisters. Their duties will consist of training and teaching probationers, and generally supervising their work and studies. At a recent meeting of the matrons of the Dublin Hospitals a resolution in favour of such appointments was passed, and it concluded in these words: 'A high standard of training is now necessitated by the passing of the State Registration Bill, and the formation of a Ministry of Health.' If the posts under the Ministry of Health are to be given to other than fully trained nurses this has little meaning."

### The Explanation.

Like myself, Dr. Peacocke is puzzled, and looks round for a probable explanation. In England, he says, no representation of the Nursing Profession has been appointed to sit on the Consultative Council of the Ministry of Health, in spite of the fact that the College of Nursing was asked to submit nominations. The same omission has been made in Ireland.

In conclusion, Dr. Peacocke gave it as his considered opinion that in the past many women who would have taken up nursing as a profession, and who are socially and educationally best fitted for such work, have refrained from so doing owing to the small number of appointments open to them. The raising of the prospects, and the enlargement of the outlook, would, he is convinced, certainly raise the standard of candidate probationers. And yet, in face of the unanswerable arguments and facts, the Trained and Registered Nurse is being deliberately scrapped, and apparently without protest. The matter, it seems to me, is one that demands vigorous and immediate Parliamentary agitation.

IERNE.

